# Analysis of Resistance to Antimicrobials and Presence of Virulence/Stress Response Genes in *Campylobacter* Isolates from Patients with Severe Diarrhoea

**DOI:** 10.1371/journal.pone.0119268

**Published:** 2015-03-17

**Authors:** Haitham Ghunaim, Jerzy M. Behnke, Idil Aigha, Aarti Sharma, Sanjay H. Doiphode, Anand Deshmukh, Marawan M. Abu-Madi

**Affiliations:** 1 Department of Health Sciences, College of Arts and Science, Qatar University, P.O. Box 2713, Doha, Qatar; 2 School of Life Sciences, University of Nottingham, University Park, NG7 2RD, Nottingham, United Kingdom; 3 Department of Laboratory Medicine and Pathology, Hamad Medical Corporation, P.O. Box 3050, Doha, Qatar; Linneaus University, SWEDEN

## Abstract

*Campylobacter* infections are a major cause of diarrhoea world-wide and two of the antimicrobials used for their control (erythromycin and ciprofloxacin) have been losing efficacy in recent years. In a sample of 174 genotyped isolates from the stools of patients with severe diarrhoea in Qatar, collected between 2005 and 2012, 63.2% showed resistance to ciprofloxacin, 8.6% to erythromycin, 0.57% to chloramphenicol and all were sensitive to gentamycin. While 33.9% of isolates were sensitive to all four antimicrobials, 59.8% were resistant to at least one, 6.3% were resistant to two and none showed resistance to three antimicrobials. There was no host sex- or age-dependence among isolates resistant to ciprofloxacin and erythromycin and no significant variation was found with the region of origin of the patients. All isolates were screened for the presence of 3 virulence factors (*ciaB*, *cadF* and *cdtB*) and two stress-response factors (*htrB* and *clpP*), all of which were present in more than 50% of the isolates. Host sex-, age- and region of origin-dependent variations in prevalence were found for some of these factors. Data analysis for the combination of virulence factors and their effect on antimicrobial resistance indicated that the prevalence of resistance to both erythromycin and ciprofloxacin was higher in isolates harbouring *ciaB* but not *clpP*. Prevalence of resistance to ciprofloxacin was similar in *clpP* positive and negative isolates also possessing *htrB*, while for *htrB*-negative isolates prevalence was higher in the absence of *clpP*. These results are discussed and their implications are highlighted.

## Introduction

*Campylobacter* spp. are the leading cause of bacterial gastroenteritis worldwide [1]. These zoonotic bacteria can colonize humans with a relatively low infective dose and have potentially life-threating sequelae. *Campylobacter* spp. are suspected of causing disease via several mechanisms such as cytotoxin production, intestinal cell invasion, and adherence and translocation. The production of cytotoxin after intestinal colonization by ingested organisms arrests the cellular cycle, ultimately leading to cell death and thereby inducing diarrhoea [2]. The ability to invade intestinal cells results in damage to the mucosal surface cells of the jejunum, ileum and colon [3]. Finally, *Campylobacter* has been reported to translocate extra-intestinally, crossing the epithelium and gaining access to other parts of the body [4].

The drug of choice for the treatment of human *Campylobacter* infections is erythromycin (member of the macrolide family), followed by ciprofloxacin (fluoroquinolone family) and tetracycline [5], however, a dramatic increase in *Campylobacter jejuni* resistance to fluoroquinolones has been observed in the USA since the mid-1990s [5]. Initially, during the period of 1982–1992, no isolates of *C*. *jejuni* showed resistance to fluoroquinolone, and only 2% were found to be resistant to erythromycin among 142 patients [5]. By 2001, resistance to fluoroquinolone had developed, and was widespread (40.5% among 47 *C*. *jejuni* isolates) [5]. This rise of resistance to fluoroquinolone in human *C*. *jejuni* isolates has been linked to the licensing of fluoroquinolone use in poultry production [6]. Enrofloxacin, a related fluoroquinolone, was licensed in the early 1990s in Europe and Asia, and soon afterwards a high incidence of resistant *C*. *jejuni* isolates was reported from animal sources, for example from Spain (99% *C*. *jejuni* isolated from broiler farms), while 72% of human isolates were found to show resistance in the same period [7]. A similar trend was observed in Taiwan, where 92% of *C*. *jejuni* isolated from chickens and 52% of human isolates were found to be resistant to fluoroquinolones by 1998–2008 [8,9]. Fluoroquinolones are just one example, as increased antimicrobial resistance has been documented also for other commonly used antimicrobials, including clindamycin, erythromycin, streptomycin, and tetracycline [10].

Several mechanisms for antimicrobial resistance have been suggested for *Campylobacter* differing between the drugs involved. For fluoroquinolones the main resistance mechanism is probably a mutation in the *gyrA* gene, which encodes part of the DNA gyrase [11], while tetracycline resistance is thought to be typically mediated through the *tetO* gene [12,13]. For macrolides, a mutation in the target ribosome binding site mediates resistance [14]. Compared with infection by antimicrobial-susceptible strains, antimicrobial-resistant strains of *Campylobacter* have been reported to be associated with a longer duration of clinical symptoms [15,16]. Ciprofloxacin-resistant *C*. *jejuni* strains colonize poultry intestinal tracts better than isogenic susceptible *C*. *jejuni* strains even in the absence of fluoroquinolone selection pressure [17], indicating an association between resistance and the ability to colonize.

In addition, several genes have been linked to enhanced virulence of *Campylobacter* but the most important ones are *Campylobacter* invasion protein B (*ciaB*), cytolethal distending toxin B (*cdtB*) which disrupts mucosal barriers by causing host cell death, *Campylobacter* adhesin to fibronectin F (*cadF)*, and the heat survival and stress-response proteins *htrB* and *clpP*. It is well-documented that there is an association between certain *Campylobacter* virulence genes and the pattern of clinical infection [18,19]. However, virulence may also be involved in modulating the expression of resistance to antimicrobial agents and such an association of antimicrobial resistance with virulence genes has already been noted in bacterial pathogens including *Escherichia coli* and *Enterococcus faecalis* [20,21] but not yet for *Campylobacter*.

To our knowledge, no data are available from Qatar regarding the status of antimicrobial resistance in *Campylobacter*. Herein, we first analyzed a nine-year dataset from the Hamad Medical Corporation (HMC) from which we extracted prevalence data on the most common enteric bacterial pathogens encountered in recent years in the stools of subjects with severe bloody diarrhoea in Qatar, in order to assess the relative importance of *Campylobacter* in the local population. We then investigated the prevalence and association of antimicrobial resistance and several virulence factors among *C*. *jejuni* isolates from symptomatic patients presenting as outpatients at the HMC hospital in order to enable knowledge based informed decisions relevant to the continuously changing dynamics of this society to be made by local health professionals and policy makers [22].

## Materials and Methods

### Clinical specimens

Ethical approval was obtained by Hamad Medical Corporation, Medical Research Center (Research protocol #13334/13). Consent was not required as the data were analyzed anonymously. All outpatients with severe and bloody diarrhoea are routinely screened at the HMC hospital for the presence of enteric bacteria using U.K. standards for microbiology investigations [23].

As part of the current work, data on the number of subjects screened and positives for each infectious agent were made available for the period 2005 to 2013, and these data are presented as prevalence values, in order to show the relative frequencies of enteric bacteria among subjects with severe diarrhoea in Qatar.

Further work, however, focused on *Campylobacter*. For this we exploited 216 cryopreserved (−80°C) specimens from earlier years as well as across the period for which we have presented prevalence values. Although we attempted to grow the bacterium from all 216 specimens known to be positive for *Campylobacter*, only 174 (*n* = 101 for 2005 to 2008 and *n* = 73 for 2009 to 2012) produced colonies of bacteria that could be genotyped and assessed for sensitivity to antimicrobials.

Specimens were tested for the presence of *Campylobacter spp*. using standard culturing techniques [24]. Specimens were streaked directly on Campyfix media supplemented with Skirrow *Campylobacter* selective antibiotic mixture (Oxiod) and incubated at 42°C under microaerobic conditions for 48 hours. The isolates were identified as *C*. *jejuni* based on the colony morphology, catalase, oxidase, hippurate hydrolysis, and H_2_S (TSI) biochemical tests, in addition to resistance to nalidixic acid and sensitivity towards cephalothin. A total of a 174 specimens identified positive for *C*. *jejuni* were analyzed. DNA was extracted directly from bacterial culture using a Mericon DNA extraction (Qiagen) kit. Specimens were confirmed to be *C*. *jejuni* by real-time PCR using a previously published primers/probe pair detecting the 16S rRNA gene [25].

### Presence of potential virulence factors genes in *Campylobacter* isolates

Real-time PCR was used to detect the presence of three of the most important *Campylobacter* virulence factors. The genes are *ciaB*, *cadF*, and *cdtB* which are involved mainly in adhesion and invasion [26]. Screening for another two genes that enhance the survival of the bacteria in the environment and the host (*htrB* and *clpP*) was performed. These five genes will be referred to as virulence factors from this point onwards. The primers (Table 1) were designed using primer design software (Primer3) based on available sequences in GenBank. The primers were tested on *C*. *jejuni* ATCC 33560 strain to confirm their efficiency. For each reaction, the following conditions were used: A total of 7μl of Sybrselect (Life Technologies) master mixture was used in each reaction along with 0.3μl primer mixture, 2.5μl specimen DNA, and 10.2μl molecular grade water. RT-PCR cycles consisted of holding for 2 minutes at 95°C, followed by 40 cycles at 95°C for 15 sec, 55°C for 35 sec, and 72°C for 1 minute. Initially, specimens having positive Ct values were confirmed by agarose gel electrophoresis for each of the genes targeted in this study. Specimens were run in duplicates.

**Table 1:** Primers for virulence genes and stress response genes used in this study.

Genes	Accession number	Primers	Sequence
*cadF*	U87559.1	Forward	5′- TAT GGT GTA GAA AAA AGT CGC ATC A-3′
		Reverse	5′- ATC CGC TCT ACC TTC TTT AGT GTC A-3′
*cdtB*	CP008787.1	Forward	5′- AAT GCA AGC TGA AGA AGT GAT TGT-3′
		Reverse	5′- AGC ATC ATT TCC ATT GCG AAT-3′
*ciaB*	AF114831.1	Forward	5′- CAA CTT TAT ATT TGC ACT CCG ATG-3′
		Reverse	5′- GGA ACG ACT TGA GCT GAG AAT AAA C-3′
*clpP*	CP008787.1	Forward	5′- TGG GAG CAT TTT TGC TTA GTT G-3′
		Reverse	5′- CTC CAC CTA AAG GTT GAT GAA TCA T-3′
*htrB*	AL111168.1	Forward	5′- CGC ACC CAA TTT GAC ATA GAA C-3′
		Reverse	5′- TTT TTA GAG CGC TTA GCA TTT GTC T-3′

### Testing for antimicrobial resistance

The susceptibility of each *C*. *jejuni* isolate to erythromycin, ciprofloxacin, gentamicin and chloramphenicol was determined using the E-test (bioMerieux, Durham USA) and read after 24 hours incubation in a microaerophilic environment at 42°C. The Minimum Inhibitory Concentrations (MIC) breakpoints of erythromycin (susceptible ≤8μg/ml), and ciprofloxacin (susceptible ≤1μg/ml) were used as recommended by Clinical Laboratory Standards Institute approved guidelines [27] for infrequently isolated or fastidious bacteria, and for gentamicin (susceptible ≤4μg/ml) and chloramphenicol (susceptible ≤8μg/ml), as recommended for non-Enterobacteriaceae [28].

### Statistical analysis

Throughout, data are presented as prevalence values (%) with 95% confidence limits (in columns in tables, in parenthesis in the text and as error bars on figures), calculated as described by Rohlf and Sokal [29], employing bespoke software. For sample sizes in excess of 1000 we used the Poisson distribution if the number of positives was 100 or fewer, and for those over 100 we used the Gaussian distribution. All calculations were carried out on numbers based to at least three decimal places, but are rounded to the nearest single decimal place or two decimal places as relevant in the text and tables.

The first data-set used for analysis comprised 26,140 subjects screened for enteric bacteria. These data were analysed using maximum likelihood on log-linear analysis of contingency tables, implemented in IBM-SPSS version 22, with YEAR (9 levels, corresponding to 2005 to 2013) and the presence/absence of each infectious agent in turn (INFECTION- present or absent) as a binary factor.

The second data-set comprised 174 isolates that were positive for *Campylobacter* and were genotyped as well as assessed for sensitivity to each of the four antimicrobials used in the study. These were derived from 104 male and 70 female subjects, for whom age was recorded in months if under 1 year-old, and then in years for all older subjects. Most came from children under three years of age (62.6%), and hence to allow analysis with age of host taken into consideration, they were allocated to 5 age classes as follows: age class 1 = children less than 1 year-old (for males *n* = 22, mean age 0.47; for females *n* = 12, mean age = 0.53); age class 2 = 1 year-old children (for males *n* = 24, for females *n* = 20); age class 3 = 2 year-old children (for males *n* = 21, for females *n* = 10); age class 4 = 3–12 year-old (for males *n* = 26, mean age = 5.2; for females *n* = 17, mean age = 6.7); age class 5 = 19–75 year-old (for males *n* = 11, mean age = 48.7; for females *n* = 11, mean age = 40.6).

The subjects were allocated to five geographical regions of origin because they came from 24 different countries as follows: Qatar (*n* = 72), Arabian Peninsula (total *n* = 33; Iran *n* = 7; Iraq *n* = 1; Jordan *n* = 4; Oman *n* = 3; Palestine *n* = 7; Saudi Arabia *n* = 1; Syria *n* = 1; Yemen *n* = 9), Asia (total *n* = 42; Afghanistan *n* = 1; Bangladesh *n* = 2; India *n* = 16; Indonesia *n* = 1; Pakistan *n* = 20; Philippines *n* = 1; Sri Lanka *n* = 1), Africa (total *n* = 21; Egypt *n* = 11; Eritrea *n* = 1; Morocco *n* = 1; Sudan *n* = 6; Tunisia *n* = 2) and elsewhere (total *n* = 6; Canada *n* = 2; United Kingdom *n* = 1; United States *n* = 3).

For analysis of presence/absence of sensitivity to specific antimicrobials, and presence/absence of virulence factors we used maximum likelihood methods on log-linear analysis of contingency tables with SEX (2 levels, male and female), AGE (5 age classes as detailed above), REGION (region of origin at 5 levels as detailed above) and PRESENCE/ABSENCE of either resistance to a specified antibiotic or of a specific virulence factor (binary factor). Full factorial models were reduced to minimum sufficient models by step-wise deletion of non-significant terms until only significant effects remained (backward selection procedure). All Chi-squared values cited refer to these significant terms in minimum sufficient models, with all other relevant factors taken into consideration.

In specific cases we also employed 2x2 and 2x*n* Chi-squared test to test specific hypotheses as indicated in the text, using the methods described previously [30].

## Results

### Prevalence of enteric bacteria in stool specimens of outpatients with diarrhoea

**Fig 1:**
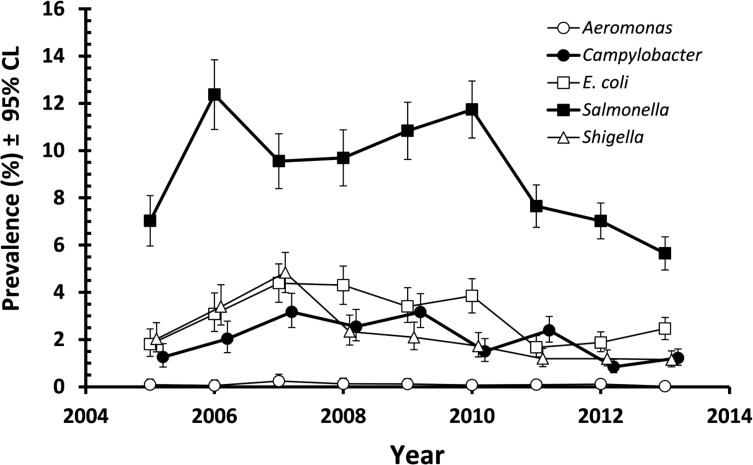
Prevalence of enteric bacteria in the stools of outpatients reporting to the MHC with severe diarrhoea in the period 2005–2013. The sample sizes for 2005–2013 were 2220, 1916, 2460, 2394, 2528, 2726, 3347, 4373 and 4176, respectively.

Fig. 1 shows the annual prevalence of pathogenic enteric bacteria in the period 2005 to 2013, in 26,140 subjects whose stools were examined because they had severe and bloody diarrhoea. The prevalence of *Salmonella* was the highest across the whole period (8.6% [8.28–8.96]) but there was significant variation between years (YEAR*INFECTION, *χ* ^2^_8_ = 161.2, *P*< 0.001). Peak prevalence was recorded in 2006 and this was followed by a consistent decline over the following years. *E*. *coli* (2.8% [2.64–3.04]), *Shigella* (2.8% [1.84–2.18]) and *Campylobacter* (1.9% [1.73–2.06]) all showed lower but similar prevalence over the whole of this period, again with some variation between years, which in all three cases was significant (*χ* ^2^_8_ = 94.9, 139.1 and 97.5, respectively and *P*<0.001 in all cases). As with *Salmonella*, there was a gradual decline in cases in the later years of the period (Fig. 1). Across the whole period, 26 cases of *Aeromonas*, 7 of *Vibrio*, and 3 of *Yersinia* were also detected. A detailed breakdown of the number of cases of *Aeromonas*, *Campylobacter*, *E*. *coli*, *Salmonella*, *Shigella*, *Vibrio* and *Yersinia* positive cases is provided as supplementary information (See S1 Table).

### Factors affecting resistance of *Campylobacter* to antimicrobials

#### Gentamycin and chloramphenicol

All specimens were sensitive to gentamycin and only one specimen showed resistance to chloramphenicol (prevalence of resistance = 0.57% [0.052–4.198]), so these were not analysed further, other than when the specimen that was resistant to chloramphenicol was included in the analysis of multiple-drug resistance.

#### Ciprofloxacin

The most resistance was observed to ciprofloxacin (63.2% [54.02–71.63]), and this was similar for isolates from both host sexes (Table 2), across all age classes (Table 2; range 59.1% to 68.2%), and among isolates from subjects from the five regions (Table 2; range 57.6% to 72.1%). No significant effects were detected for the effect of SEX, AGE or REGION of origin, or any interactions between these factors.

**Table 2:** The effect of host sex, age and country of origin on resistance of *Campylobacter* isolates to antimicrobials.

Class		Ciprofloxacin		Erythromycin	
	*n*	%	95% CL	%	95% CL
Males	104	61.5	54.43–68.16	7.7	4.65–12.40
Females	70	65.7	54.14–75.91	10.0	4.82–19.14
Age class 1	34	64.7	49.84–77.54	11.8	4.92–24.50
Age class 2	44	59.1	41.97–74.63	6.8	1.53–20.36
Age class 3	31	67.7	53.55–79.82	9.7	3.77–21.51
Age class 4	43	60.5	43.58–75.58	7.0	1.65–20.37
Age class 5	22	68.2	45.35–84.82	9.1	1.64–29.07
Qatari	72	59.7	47.95–70.83	11.1	5.45–20.71
Arabian Peninsula	33	57.6	42.87–71.27	3.0	0.39–12.95
Asia	42	73.8	57.28–86.05	7.1	1.78–20.39
Africa	21	61.9	40.33–80.26	14.3	4.01–35.43
Elsewhere	6	66.7	27.14–93.71	0	0–41.13

#### Erythromycin

Resistance to erythromycin was rarer (8.6% [4.57–15.32]), also similar for isolates from both sexes (Table 2) and across all five age classes (Table 2; range 7.0% to 11.8%). However, there were some discrepancies between regions of origin of subjects (Table 2; 0% to 14.3%), but these were not significant.

### Multiple drug resistance

While 33.9% [25.81–43.10] of specimens were sensitive to all four antimicrobials, 59.8% [50.55–68.54] were resistant to at least one and 6.3% [3.07–12.27] were resistant to two. None showed resistance to 3 or all four antimicrobials.

### Factors affecting prevalence of virulence factors

All five virulence factors were present in more than 50% of specimens (Table 3). The most frequent was *cdtB* and the least frequent *cadF*. Table 3 shows prevalence by geographic origin of the hosts.

**Table 3:** Prevalence of virulence factors among isolates from subjects from different geographical regions and in the combined dataset.

Region of origin	*ciaB*		*cadF*		*cdtB*		*htrB*		*clpP*	
	%	95% CL	%	95% CL	%	95% CL	%	95% CL	%	95% CL
Qatar	76.4	64.99–85.14	63.9	52.14–74.33	90.3	81.02–95.43	56.9	45.16–68.07	81.9	71.28–89.43
Arabian Peninsula	66.7	52.03–79.31	51.5	36.83–66.11	87.9	75.31–94.77	87.9	75.31–94.77	84.8	71.35–93.03
Asia	71.4	54.87–84.12	52.4	35.77–68.22	83.3	67.34–92.65	71.4	54.87–84.12	92.9	79.61–98.22
Africa	61.9	40.33–80.26	61.9	40.33–80.26	81.0	59.68–93.21	57.1	35.44–76.73	66.7	44.90–84.10
Elsewhere	83.3	41.14–99.14	100	58.87–100	100	58.87–100	100	58.87–100	83.3	41.14–99.14
Combined	71.8	62.98–79.36	59.8	50.55–68.54	87.4	80.12–92.48	67.8	58.64–75.80	83.3	75.34–89.21

**Fig 2:**
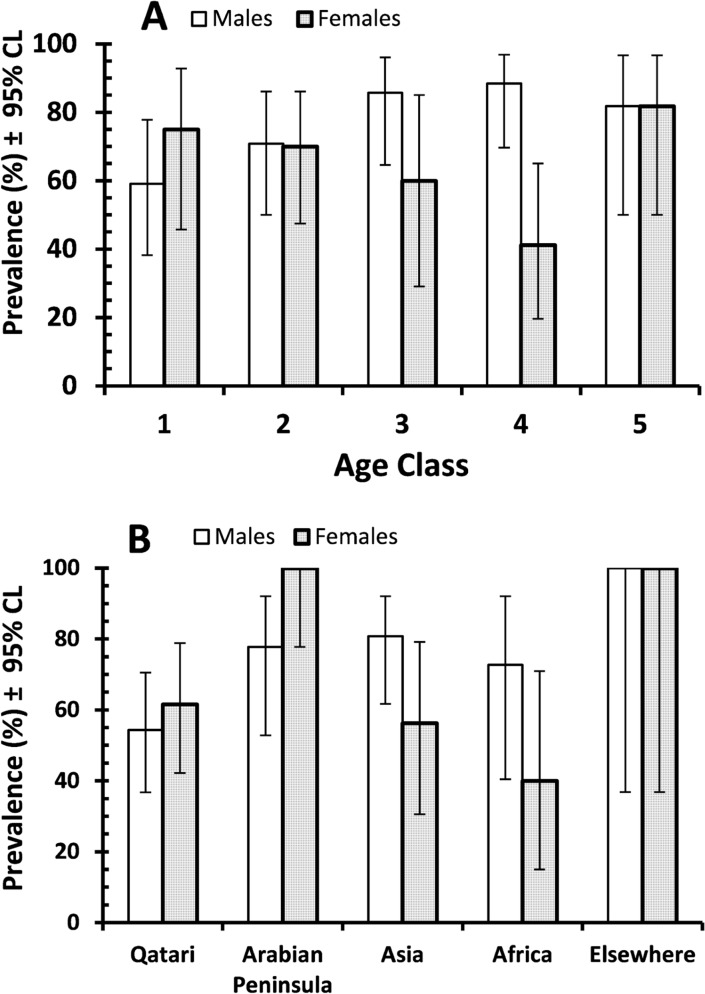
Prevalence of the virulence factor *ciaB* (A) and *htrB* (B) among male and female subjects in each of the age classes (A) and from each of the five regions of origin used for analysis (B). The sample sizes in A were as follows: for the 104 male subjects, 22, 24, 21, 26 and 11, and for the 70 female subjects 12, 20, 10, 17 and 11 for age classes 1–5 respectively in both cases. For the age span and average age of each age class see Materials and Methods. The sample sizes in B were as follows: for the 104 male subjects, 46, 18, 26, 11 and 3, and for the 70 female subjects 26, 15, 16, 10 and 3 for the five regions of origin (Qatar, Arabian Peninsula, Asia, Africa and elsewhere) respectively in both cases. For further details of countries of origin in each region see Materials and Methods.

For *ciaB* there was a significant interaction between SEX and AGE on prevalence (*χ* ^2^_4_ = 10.8, *P* = 0.029), as illustrated in Fig. 2-A. The prevalence of *ciaB* increased with the age of males, but among females prevalence was lower in age classes 3 and 4. There was no difference between isolates from subjects from different regions.

The prevalence of *cadF* only differed between the sexes (males = 67.3% [60.24–73.68]; females = 48.6% [36.94–60.23];*χ* ^2^_1_ = 6.09, *P* = 0.014), and there was no effect of AGE or REGION of origin (Table 3) and no interactions between these factors.

None of the factors affected the prevalence of *cdtB* among the specimens, but there was a significant interaction between SEX and REGION of origin on the prevalence of *htrB* (Fig. 2-B; *χ* ^2^_4_ = 10.7, *P* = 0.03). While in Qatar and the Arabian peninsula prevalence of *htrB* showed female bias, in Asia and Africa the bias was in favour of the male subjects.

The prevalence of *clpP* also varied markedly between sexes with a male bias (males = 89.4% [84.24–93.20]; females = 74.3% [63.05–83.16];*χ* ^2^_1_ = 6.8, *P* = 0.009) and there was a significant interaction between AGE and REGION (*χ* ^2^_16_ = 29.3, *P* = 0.022), which we did not explore further.

### Relationship between virulence factors and resistance to ciprofloxacin and erythromycin

Although the data in Table 4 show some bias in prevalence of virulence factors in some cases, none of these were significant and therefore none of the virulence factors were clearly linked to resistance to either drug. The nearest to significance was the discrepancy in the prevalence of *htrB* among ciproxin sensitive and resistant isolates, for which there was a difference of 13.3% between resistant isolates compared with those that were sensitive (Table 4). However, analysis revealed that this was just the wrong side of the cut-off for significance, so this is indicative of a link but is not conclusive.

**Table 4:** Prevalence of virulence factors among erythromycin sensitive and resistance isolates, and among ciprofloxacin sensitive and resistant isolates.

Virulence factor	Erythromycin				Ciprofloxacin			
	Sensitive (*n* = 159)		Resistant (*n* = 15)		Sensitive (*n* = 64)		Resistant (*n* = 110)	
	%	95% CL	%	95% CL	%	95% CL	%	95% CL
*ciaB*	71.7	63.24–78.90	73.3	46.58–90.33	67.2	56.10–76.73	74.5	67.78–80.41
*cadF*	61.0	52.25–69.02	46.7	22.23–70.60	54.7	43.54–65.27	62.7	55.44–69.51
*cdtB*	88.1	81.26–92.69	80.0	53.43–94.31	89.1	80.12–94.49	86.4	80.64–90.73
*htrB*	69.2	60.55–76.73	53.3	29.40–77.77	59.4*	**48.25–69.92**	**72.7**	**65.73–78.77**
*clpP*	84.9	77.62–90.32	66.7	39.68–85.83	85.9	76.31–92.31	81.8	75.53–86.85

*2x2 Chi-squared test, *χ* ^2^_1_ = 3.3, *P* = 0.059

### Relationship between virulence factors and isolates of *Campylobacter* showing sensitivity or resistance to 1 or 2 antimicrobials

Table 5 summarises the prevalence of each virulence factor among the isolates that were either sensitive to any one or two of the antimicrobials. No isolate was resistant to three antimicrobial and no significant effects were detected.

**Table 5:** Prevalence of virulence markers among isolates which are sensitive or resistant to 1 or 2 antimicrobials.

Virulence factor	Sensitive to all antimicrobials (*n* = 59)		Resistant to 1 antimicrobials (*n* = 104)		Resistant to 2 antimicrobials (*n* = 11)		Statistical analysis
	%	95% CL	%	95% CL	%	95% CL	
*ciaB* +ve	67.8	57.11–76.98	73.1	66.27–78.96	81.8	50.00–96.66	χ22 = 0.82; P = 0.66 = NS
*ciaB*—ve	32.2	23.02–42.89	26.9	21.04–33.73	18.2	3.34–50.00	
*cadF* +ve	55.9	45.19–66.13	62.5	55.39–69.13	54.5	26.46–80.04	*χ* ^2^_2_ = 0.742; *P* = 0.69 = NS
*cadF*-ve	44.1	33.87–54.81	37.5	30.87–44.61	45.5	19.96–73.54	
*cdtB* +ve	89.8	81.58–94.80	86.5	80.99–90.78	81.8	50.00–96.66	*χ* ^2^_2_ = 0.44; *P* = 0.80 = NS
*cdtB*—ve	10.2	5.20–18.42	13.5	9.22–19.01	18.2	3.34–50.00	
*htrB* +ve	61.0	50.30–70.76	72.1	65.28–78.06	63.6	33.29–86.49	*χ* ^2^_2_ = 2.16; *P* = 0.34 = NS
*htrB*—ve	39.0	29.24–49.70	27.9	21.94–34.72	36.4	13.51–66.71	
*clpP* +ve	86.4	77.47–92.37	83.7	77.76–88.37	63.6	33.29–86.49	*χ* ^2^_2_ = 2.45; *P* = 0.25 = NS
*clpP*-ve	13.6	7.63–22.53	16.3	11.63–22.24	36.4	13.51–66.71	

All statistical tests here were 2x3 Chi-squared tests

### Combinations of virulence factors and their effects on resistance to ciprofloxacin and erythromycin

We first examined pairwise combinations of the five virulence factors and their effects on resistance to both antimicrobials. Of the ten possible combinations, in the case of erythromycin prevalence of resistance was lower in those showing both factors compared with those with neither factor in eight out of ten cases, but the degree of reduction in prevalence of resistance ranged only from −1.9% (*ciaB* and *htrB*) to −10.3% (*cadF* and *htrB*). In two cases prevalence of resistance was marginally higher in those isolates expressing both virulence factors (*ciaB* and *cdtB* by 7.1%; *ciaB* and *clpP* by 5.4%), but in no case was the difference in prevalence of resistance among isolates that were negative for both factors and those expressing both, significant. Six combinations showed increased resistance to ciprofloxacin, ranging from 7.5% (*cadF* and *clpP*) to 19.2% (*cadF* and *htrB*), the latter being just outside the cut-off for significance (2x2 Chi-squared test, *χ* ^2^_1_ = 3.47, *P* = 0.062). Four combinations showed lower resistance ranging from −4.5% (*ciaB* and *cdtB*) to −15.7% (*cdtB* and *clpP*) and none were significant.

**Fig 3:**
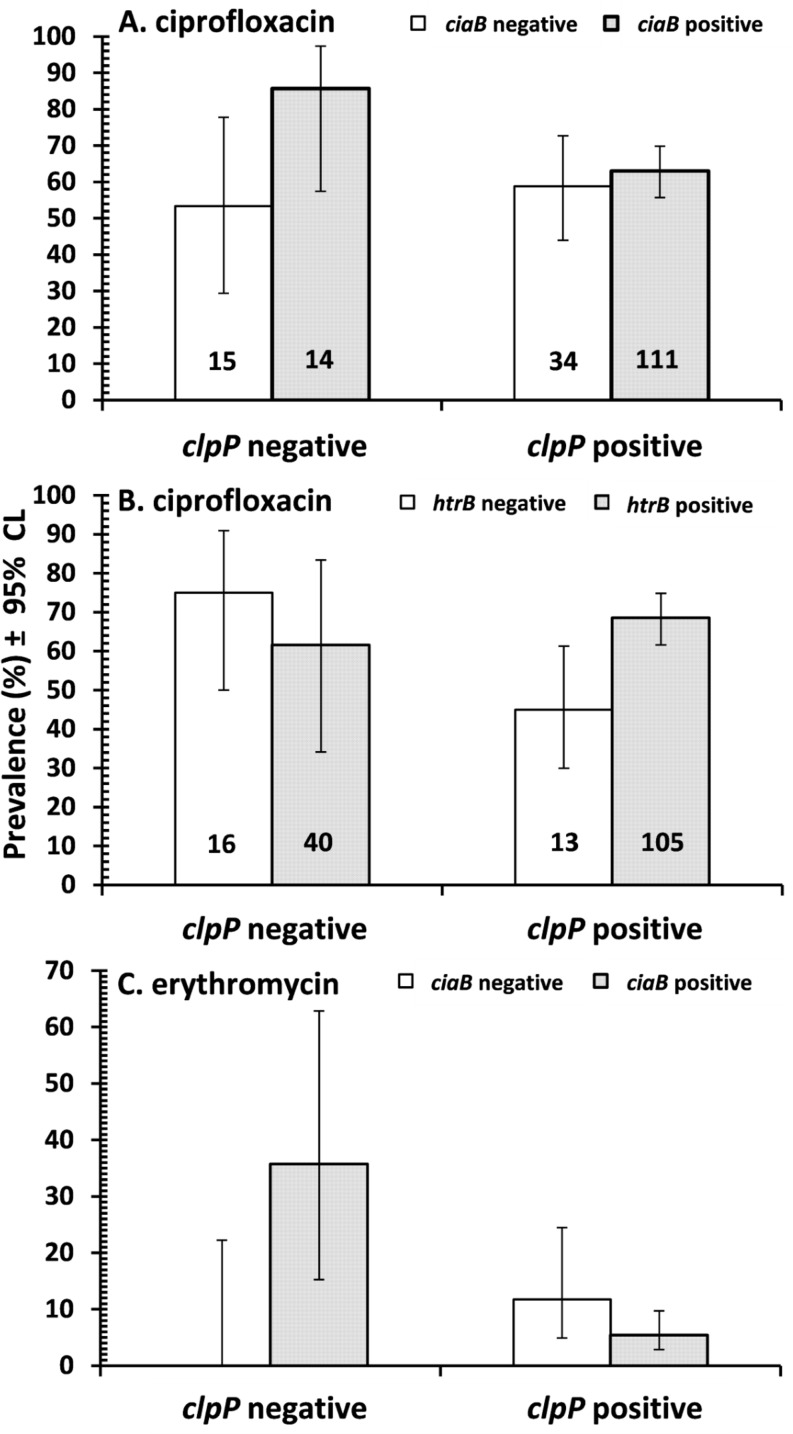
Prevalence of resistance to ciprofloxacin (A and B) and erythromycin (C) in *Campylobacter* isolates which were positive or negative for the *clpP* virulence factor, in the presence/absence of *CiaB* (A and C) and *htrB* (B). Sample sizes are given on the columns in A and B, and for C the sample sizes were the same as in A.

Analysis by fitting log-linear models indicated three significant interactions. Fig. 3-A shows that prevalence of resistance to ciprofloxacin was very similar in *ciaB* negative isolates whether they expressed *clpP* or not, while for *ciaB* positive isolates prevalence of resistance was higher in the absence of *clpP* (*χ* ^2^_1_ = 5.2, *P* = 0.023). Fig. 3-B shows that while in *clpP* negative isolates prevalence of resistance to ciprofloxacin was similar whether they expressed *htrB* or not, among *clpP* positive isolates prevalence of resistance was higher when *htrB* was also present (*χ* ^2^_1_ = 6.9, *P* = 0.002). Finally in Fig. 3-C it can be seen that among *clpP* negative isolates prevalence of resistance to erythromycin was higher in *ciaB* positive isolates, while in *clpP* positive isolates prevalence of resistance was generally lower and only differed marginally between isolates that were positive or negative for *ciaB* (*χ* ^2^_1_ = 9.5, *P* = 0.002).

## Discussion

Diarrhoeal diseases remain a leading cause for morbidity and mortality worldwide despite advances in our understanding of pathogen transmission patterns. The nine-year dataset we analyzed from HMC for 26,140 subjects who presented with clinical gastrointestinal signs showed that a group of bacterial pathogens are responsible for a significant proportion of these cases. Indeed, *Salmonella*, *Campylobacter*, *E*. *coli*, or *Shigella* were involved collectively in at least 15% of the cases. Prevalence of these pathogens varied over the years, with a clear decline in most cases towards the end of the study period. This year-to-year variation in prevalence emphasizes the importance of the surveillance program HMC is implementing to monitor this group of pathogens. The analysis of the nine-year data set also confirmed the importance of *C*. *jejuni* as a common enteric pathogen in Qatar.

As the next step in this study, we investigated the prevalence of antimicrobial resistance and its association with the presence of virulence and environment stress response genes in *C*. *jejuni* isolates from symptomatic individuals. As erythromycin is the drug of choice for the treatment of *C*. *jejuni*, it was reassuring to find that antimicrobial resistance to this drug was relatively low, as only 8.6% of the isolates were resistant. On the other hand, the relatively high level of resistance to ciprofloxacin (63.2%), regardless of the country of origin of the patient, is alarming as this fluoroquinolone is indicated for the treatment of a variety of critical infections in adults [31]. Fluoroquinolones are considered critical drugs for the treatment of humans by the World Health Organization and the veterinary fluoroquinolones, enrofloxacin and danofloxacin, are commonly used antimicrobial drugs in food-producing animals and are thought to play a role in the spread of resistance to human isolates [32,33]. A high rate of resistance has also been reported in the United Arab Emirates where 85.4% of isolates were resistant to this antimicrobial [34], while isolates from other countries have shown lower rates of prevalence, e.g. 40% in Poland and 2% in Australia were reported to be resistant [35,36]. As the use of fluoroquinolones in food-producing animals is permitted in the UAE and Qatar, while it is banned in Australia, this difference in the levels of resistance could be attributable to the continued use of these antimicrobials. Further assessment of the rate of antimicrobial resistance among *C*. *jejuni* isolates from animals, in addition to concurrent genetic relationships, should determine if this is truly the case in Qatar.

As for the presence of virulence factors, all five factors investigated here were present in more than 50% of the specimens, with *cdtB* being the most frequently encountered and *cadF* the least. *cadF* is an adhesin and fibronectin-binding protein involved in the process of invasion by inducing structural re-arrangement through microfilament manipulation [37]. Since all the *Campylobacter* isolates investigated here were obtained from symptomatic patients and only 59.8% carried this gene, it is likely that the presence or absence of this one gene is not enough to set up infection or abolish it. The low prevalence of *cadF* contrasts with several other studies that have found this virulence factor in nearly all of the *Campylobacter* isolates that were examined [38,39,40,41]. *Campylobacter* strains that have a *cadF* deletion mutation are unable to colonize in chickens and their internalization ability into INT 407 intestinal cells is compromised [37]. However, clearly isolates in which we did not detect the gene survive in human subjects, as evidenced in our study, but because of the methods employed here we cannot eliminate the possibility that a variant of the gene, that was not detected by our PCR based methodology, was present and capable of mediating invasion of the host (see below for further discussion). Nevertheless, there is no current information on any variants of this gene, with a different nucleotide base sequence, that would not have been detected by our PCR primers.

The cytotoxin produced by *C*. *jejuni* can damage nuclear DNA, causing the cell cycle to arrest in the G2 or M phase [42]. Cytolethal distending toxin is also involved in the production of pro-inflammatory cytokines such as IL-8 from intestinal epithelial cells *in vitro* [43] which might explain the role it plays in pathogenesis. Several studies have indicated that the prevalence of *cdtB* in isolates from poultry or humans exceeds 90% [41], which is in agreement with our findings and was expected, given that the isolates studied here were all from clinical cases.

The *ciaB* gene is critical for invasion of epithelial cells as mutants lacking this gene are not able to invade cell lines *in vitro* [44]. As *C*. *jejuni* lacks a type III secretion system, this protein is secreted through a flagellar export system [45], but again in this study we did not detect *ciaB* in 28.2% of the isolates studied. Although *ciaB* was shown to be important for invasion using *in vitro* systems, in this study human isolates that lack this gene still caused clinical signs, indicating that even in its absence *C*. *jejuni* can cause disease.

*C*. *jejuni* is likely to encounter a wide range of temperatures during a contamination cycle and must therefore be able to adapt and respond to these temperatures. In contrast to other foodborne pathogens, *C*. *jejuni* does not possess genes encoding cold-shock proteins such as *cspA* which might explain why it cannot grow at low temperatures [1,46]. On the other hand, *C*. *jejuni* utilizes heat-shock proteins, including the ATP-dependent proteases *clpP*, which repair and prevent damage caused by accumulation of unfolded proteins [47,48]. Our study detected an interesting interaction between the presence/absence of this stress response gene and *ciaB*. In the case of resistance to both ciprofloxacin and erythromycin, isolates that did not express *clpP*, but were positive for *ciaB*, showed a higher prevalence of resistance. How the gene products of these two genes interact to bring this interaction about is not known, but it is clearly of interest if we are to achieve a comprehensive understanding of the mechanisms of the evolution and maintenance of antimicrobial resistance in *Campylobacter* infections.

The *C*. *jejuni htrB* gene encodes an acyltransferase that contributes to lipid A synthesis [49,50] and is conserved in *C*. *jejuni*. It is similar to the *htrB* gene of *Salmonella typhimurium*, *E*. *coli* and *Haemophilus influenzae*. The high prevalence of this gene in this group of clinical specimens might indicate its importance for the survival of this bacterium inside the host, but, nevertheless a proportion (32.2%) of our isolates did not express this gene, and here also we detected an interaction with *clpP* on prevalence of resistance to ciprofloxacin. While *htrB* positive isolates showed similar prevalence of resistance irrespective of whether they expressed *clpP* or not, among *htrB* negative isolates resistance was higher in the absence of *clpP*. Again, this suggests complex underlying background genetic and epistatic interactions.

Whilst we did not find any evidence of significant variation in the extent of resistance to ciprofloxacin or erythromycin that was host age-, sex- or region of origin-dependent, we did find that the presence of some of the virulence and stress factors on isolates was dependent on these host factors. For example, the prevalence of isolates expressing *ciaB* increased with the age in male subjects, but among females the prevalence was lower in subjects aged 2–12 year-old. A sex effect was also noticed for *cadF* which was more prevalent in males than females. As *cadF* is involved in adhesion to the intestinal wall of the host while *ciaB* is thought to be involved in cellular invasion of *Campylobacter*, it is likely that the different sex and age effects are linked to the different habitats for the bacteria in hosts of varying age and sex. Moreover, these sex- and age-linked effects on the expression of virulence/stress factors may in part explain the known predominance of *Campylobacter* among male subjects, which has been attributed to physiological differences between the sexes [51].

Although the findings in this study show a high prevalence of some of the virulence factors investigated, such as *cdtB* and *ciaB*, it is important to emphasize that the presence or absence of these genes is indicative, but may not predict precisely how virulent a particular *Campylobacter* isolate may be *in vivo* and further studies have to be carried out to reach a definitive conclusion. As already intimated above, it is also important to be aware that a negative result by RT-PCR could be attributed not just to an absence of the gene in question but possibly also to sequence variation at the primer binding site, perhaps indicating a variant of the same molecule with either identical or perhaps different biological activity. This is an important limitation of the molecular methods employed here, relying entirely on the amplification of the targeted region, and therefore the data should be interpreted carefully.

Positive and negative associations between virulence genes and antimicrobial resistance have been found in several bacterial pathogens including *E*. *coli* and *E*. *faecalis* [21,52]. A mutation in the *gyrA* gene in *C*. *jejuni* on the other hand enhances its ability to colonize chickens and therefore survival [17]. Another clear example is the effect of streptomycin resistance on the growth of *Salmonella*, as resistant strains have poor growth on rich media because they cannot express the *rpoS* gene which is important for regulation of environmental stress responses [53]. As the presence of antimicrobial resistance and potential virulence factors are both important in their own right, further investigation of the basis of synergistic and antagonistic interactions between their presence/absence in clinical isolates might provide new insight into the nature of *Campylobacter* pathogenesis. Indeed, methicillin resistance of *Staphylococci*, for example, has been used over the years as a marker to classify the different isolates as it is a critical factor in predicting not only the outcome of infection but also the fitness level of the isolate [54]. As for *E*. *coli*, a positive relationship has been established between multiple drug resistance and the severity and duration of clinical symptoms [55].

Finally, in this study we have provided for the first time data on the prevalence of resistance to two of the most popular and widely used antimicrobials for the treatment of *Campylobacter*, among isolates of this bacterium from subjects in Qatar. We have also quantified the frequency of five key virulence factors and detected some interactions between these and the expression of antimicrobial resistance. The exact nature of the underling mechanisms and the significance of these effects and their various interactions for the adaptability of *C*. *jejuni* is not yet clear. In view of the importance of *Campylobacter* as a disease causing organism in humans, and its frequent occurrence among the Qatari population, further work is warranted urgently and we hope to have provided here a springboard for such research in the years ahead.

## Supporting Information

S1 Table Number of cases of pathogenic enteric bacteria during the period of 2005 to 201 (XLSX)
